# Quality of Large Language Model Responses to Radiation Oncology Patient Care Questions

**DOI:** 10.1001/jamanetworkopen.2024.4630

**Published:** 2024-04-02

**Authors:** Amulya Yalamanchili, Bishwambhar Sengupta, Joshua Song, Sara Lim, Tarita O. Thomas, Bharat B. Mittal, Mohamed E. Abazeed, P. Troy Teo

**Affiliations:** 1 Robert H. Lurie Comprehensive Cancer Center, Department of Radiation Oncology, Northwestern Memorial Hospital, Northwestern University Feinberg School of Medicine, Chicago, Illinois

## Abstract

**Importance:**

Artificial intelligence (AI) large language models (LLMs) demonstrate potential in simulating human-like dialogue. Their efficacy in accurate patient-clinician communication within radiation oncology has yet to be explored.

**Objective:**

To determine an LLM’s quality of responses to radiation oncology patient care questions using both domain-specific expertise and domain-agnostic metrics.

**Design, Setting, and Participants:**

This cross-sectional study retrieved questions and answers from websites (accessed February 1 to March 20, 2023) affiliated with the National Cancer Institute and the Radiological Society of North America. These questions were used as queries for an AI LLM, ChatGPT version 3.5 (accessed February 20 to April 20, 2023), to prompt LLM-generated responses. Three radiation oncologists and 3 radiation physicists ranked the LLM-generated responses for relative factual correctness, relative completeness, and relative conciseness compared with online expert answers. Statistical analysis was performed from July to October 2023.

**Main Outcomes and Measures:**

The LLM’s responses were ranked by experts using domain-specific metrics such as relative correctness, conciseness, completeness, and potential harm compared with online expert answers on a 5-point Likert scale. Domain-agnostic metrics encompassing cosine similarity scores, readability scores, word count, lexicon, and syllable counts were computed as independent quality checks for LLM-generated responses.

**Results:**

Of the 115 radiation oncology questions retrieved from 4 professional society websites, the LLM performed the same or better in 108 responses (94%) for relative correctness, 89 responses (77%) for completeness, and 105 responses (91%) for conciseness compared with expert answers. Only 2 LLM responses were ranked as having potential harm. The mean (SD) readability consensus score for expert answers was 10.63 (3.17) vs 13.64 (2.22) for LLM answers (*P* < .001), indicating 10th grade and college reading levels, respectively. The mean (SD) number of syllables was 327.35 (277.15) for expert vs 376.21 (107.89) for LLM answers (*P* = .07), the mean (SD) word count was 226.33 (191.92) for expert vs 246.26 (69.36) for LLM answers (*P* = .27), and the mean (SD) lexicon score was 200.15 (171.28) for expert vs 219.10 (61.59) for LLM answers (*P* = .24).

**Conclusions and Relevance:**

In this cross-sectional study, the LLM generated accurate, comprehensive, and concise responses with minimal risk of harm, using language similar to human experts but at a higher reading level. These findings suggest the LLM’s potential, with some retraining, as a valuable resource for patient queries in radiation oncology and other medical fields.

## Key Points

**Question:**

How does the quality of responses from an artificial intelligence large language model (LLM) in radiation oncology compare with established sources, and what are the best metrics for evaluation?

**Findings:**

In this cross-sectional study using a Likert scale to evaluate 115 radiation oncology questions from 4 professional society websites, the LLM’s responses to the questions were on par or superior in 94% of cases for accuracy, 77% of cases for completeness, and 91% of cases for conciseness, with 2 potentially harmful responses. Key metrics included cosine similarity and readability levels, which were higher than professional responses.

**Meaning:**

Although the LLM showed high accuracy and completeness in radiation oncology queries, its higher-than-recommended readability levels suggest the need for refinement for improved patient accessibility and understanding.

## Teaser

This cross-sectional study compares the quality of answers from a large language model (LLM) with those from established sources to radiation oncology patient care questions using domain-specific and domain-agnostic metrics.

## Introduction

Artificial intelligence (AI) large language model (LLM) chatbots have shown promise in answering medical test questions,^1,2^ simplifying radiology reports,^3^ and searching for cancer information.^4^ These LLM chatbots have the potential to alter medical practice and improve efficiency by reducing workload and optimizing performance.^5^ In medical applications, the reliability of the LLM’s training data and processes is a critical concern due to the potential for factually inaccurate responses, known as “hallucinations.”^6,7^ Evaluating response quality is essential for ensuring patient safety and remains a key implementation challenge.

Radiation oncology is an intricate field of treatment that relies on specialized machinery and involves interdisciplinary expertise encompassing clinical medicine, physics, and dosimetry. During initial consultations, approximately one-third of the words used consist of medical jargon, while another one-third includes common words that may have distinct meanings in the context of radiation treatments.^8^ The complex processes involved can be intimidating or overwhelming to patients and can lead to patient anxiety, poor understanding, and difficulty in making treatment decisions and adhering to treatments. Patients often have many follow-up questions involving the treatment process, side effects, and safety, and how treatments are designed and delivered.^9^ These factors collectively contribute to the complexities and challenges associated with achieving effective patient-physician communication. In addition, cancer treatments, particularly in breast, gynecologic, and prostate cancers, can affect body image and sexual health. Patients often feel uncomfortable discussing these sensitive topics with clinicians, leading to inadequate sexual health care.^10,11,12^ Patient communication with a nonsentient LLM chatbot can lower these barriers.

The radiation oncology team often faces time constraints due to tasks such as electronic health record documentation and managing insurance authorizations.^13^ With increasing virtual care, patient messages add substantially to staff hours, potentially exacerbating physician burnout and affecting care quality.^14,15^ While preconsultation educational materials and patient-physicist consultations are proposed solutions,^16,17^ existing online resources may not adequately address specific patient queries in radiation oncology and often exceed recommended complexity levels.^18,19^

The growing use of electronic communication in health care suggests a potential role for LLM chatbots in enhancing patient-clinician interactions. However, given that the LLM was not explicitly trained for oncology-related inquiries, its ability to provide accurate, complete, and safe responses to radiation treatment questions remains unverified, highlighting a gap in current research.

Our work aims to evaluate the potential of using the LLM to answer questions commonly encountered in an initial consultation between a patient and a radiation oncology physician. As many common patient questions are found on websites and training materials provided by professional society websites, the performance of the LLM and its responses can be compared with information provided by these sites. A quantitative evaluation involving both domain-specific expertise and domain-agnostic metrics will be introduced. Although domain-specific metrics rely on expert guidance and human-in-the-loop evaluation to assess the quality, accuracy, and potential harm of the LLM’s responses, computationally generated domain-agnostic metrics are automatically computed based on statistical analysis of the text in the LLM’s responses. This combination of expert-driven and data-driven evaluation approaches provides a comprehensive assessment of the LLM’s performance for these tasks.

## Methods

The Northwestern University institutional review board deemed this cross-sectional study exempt per 45 CFR §46; informed consent was not required because it was not human participants research. This study followed the Strengthening the Reporting of Observational Studies in Epidemiology (STROBE) reporting guideline.^20^

### Question-Answer Database

Question-answer resources from the websites of 4 large oncology and radiation oncology groups were assessed. These included RadiologyInfo.org, sponsored by the Radiological Society of North America (RSNA) and the American College of Radiology (ACR); RTAnswers.org from the American Society for Radiation Oncology (ASTRO)^21^; Cancer.gov from the National Cancer Institute (NCI) at the National Institutes of Health (NIH)^22^; and Cancer.net from the American Society of Clinical Oncology (ASCO)^23^ (accessed February 1 to March 20, 2023). We adhered to all relevant professional and institutional ethical guidelines for the usage of public data as stipulated by the respective professional societies. These 4 resources were assessed by a group of radiation oncologists and radiation physicists. No single resource was found to have a comprehensive list of common questions and answers. Cancer.gov was found to have the most general radiation oncology questions and answers, and RadiologyInfo.com was found to have the most cancer subsite–specific and modality-specific questions and answers.

The common patient questions retrieved from Cancer.gov and RadiologyInfo.com were divided into 3 thematic categories: general radiation oncology, treatment modality–specific, and cancer subsite–specific questions. A database was compiled to include 29 general radiation oncology questions from Cancer.gov; 45 treatment modality–specific questions and 41 cancer subsite–specific questions from RadiologyInfo.com. Questions were then entered into the LLM chatbot ChatGPT version 3.5 (OpenAI), accessed February 20 to April 20, 2023, and answers were generated. The exact wording from Cancer.gov and RadiologyInfo.com was input into the LLM, except in cases where information subheadings on the websites were not provided in a question format. Expert answers provided by professional society online resources and the LLM-generated answers were compiled into a survey.

### Statistical Analysis

#### Domain-Specific Metrics

A Turing test–like approach was used to compare the quality of the LLM-generated responses with expert answers. The LLM-generated responses were assessed for relative factual correctness, relative completeness, and relative conciseness and organization by 3 radiation oncologists and 3 radiation physicists.^24^ A 5-point Likert scale (1: “much worse,” 2: “somewhat worse,” 3: “the same,” 4: “somewhat better,” and 5: “much better”) was used to evaluate the degree of agreement for the 3 evaluation metrics. A fourth metric, potential harm, was also evaluated using a 5-point Likert scale (0: “not at all,” 1: “slightly,” 2:“moderately,” 3: “very,” and 4: “extremely”). The ranking of each evaluator was assessed, and a mean score and SD were calculated for each metric. The mean response of the 6 raters for potential harm as well as correctness, completeness, and conciseness was plotted for each thematic category including general issues, treatment modality, and treatment site, and for subcategories within the treatment modality and treatment site categories. Given the nature of our comparative analysis, it was essential for the raters to be aware of the source of each response, whether from the LLM or professional societies, to ensure informed evaluations. The primary objective of our study was to assess the quality and reliability of the LLM’s responses in relation to expert content (ie, the ground truth), rather than testing their indistinguishability from professional advice, thus blinding was not used. Methodological rigor was maintained through multiple raters and established evaluation criteria to minimize potential bias.

Expert answers and the LLM-generated answers were then compared using cosine similarity, a computational similarity measure. Cosine similarity is used to measure the similarity of subject matter between 2 texts independent of the length of the text. A measure of 1 indicated the highest similarity, whereas 0 indicated no similarity. The Augmented Sentence Bidirectional Encoder Representations from Transformers (sentence transformers) package was used to encode the answers for processing.^25^ The similarity between encoded answers were analyzed in Python version 3.12 (Python Software Foundation).

#### Domain-Agnostic Metrics

To assess the readability of the content, a readability analysis was performed using 10 major readability assessment scales commonly used to evaluate the readability of medical literature. These 10 numeric scales included the Flesch Reading Ease, New Fog Count,^26^ Flesch-Kincaid Grade Level,^27^ Simple Measure of Gobbledygook,^28^ Coleman-Liau Index,^29^ Gunning Fog Index,^30^ FORCAST Formula,^31^ New Dale-Chall,^32^ Fry Readability,^33^ and Raygor Readability Estimate.^34^ A combined readability consensus score, which correlates with the grade level, was determined from these 10 scales. Three additional analyses of word count, lexicon, and syllable count were performed for each expert and LLM-derived answer. Mean scores were compared using a 2-sample *t* test, performed in Excel version 2310 (Microsoft Corporation). We predetermined an overall statistical significance level, with 2-sided hypothesis tests set at *P* < .05. A correction method was not applied to adjust the significance threshold for subgroup analyses; hence, findings are considered exploratory. The combination of domain-specific metrics evaluated by human experts and domain-agnostic metrics generated by automated software, along with statistical analysis, was used to systematically assess and address any inaccuracies and biases in the responses generated by the LLM. A qualitative analysis of the language used by the LLM was conducted to identify potential biases, including gender, racial, or treatment modality biases, but no significant patterns of bias were found. Statistical analysis was performed from July to October 2023.

## Results

**Figure 1.**
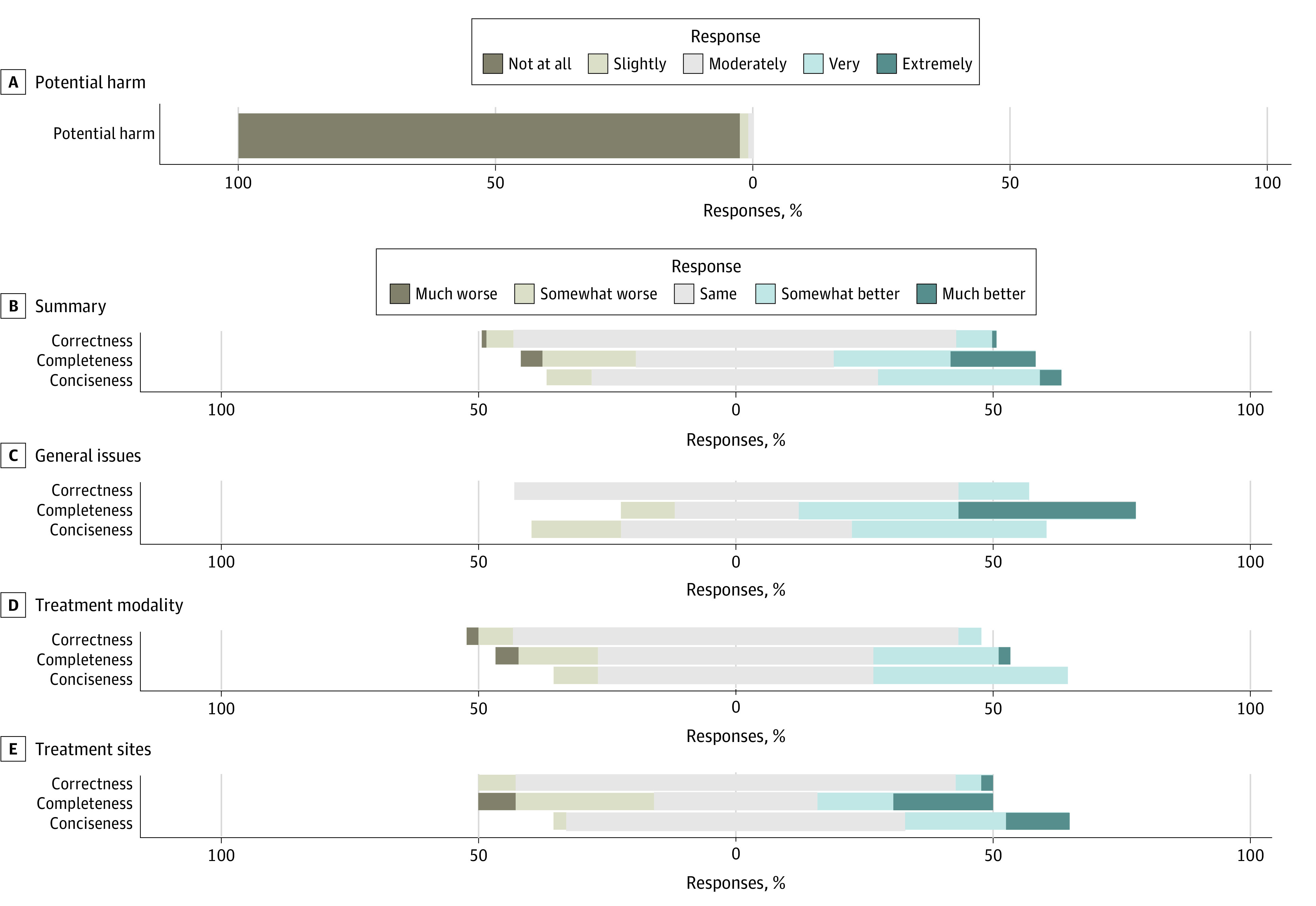
Large Language Model (LLM) Potential Harm Ratings and Comparison of LLM vs Radiation Oncology Expert Responses for 115 Questions Likert scale plot including potential harm ratings for all 115 LLM-generated responses (A); followed by comparisons of the LLM’s responses to expert answers from online resources, evaluating relative factual correctness, completeness, and conciseness, across all questions (B), general radiation oncology topics (C), treatment modality–specific issues (D), and treatment site-specific queries (E).

Out of 115 radiation oncology questions retrieved from 4 professional society websites, 113 (99%) of ChatGPT (the LLM) responses posed no potential harm; 2 LLM responses were ranked as having potential harm (Figure 1A). Potential harm was ranked moderate for 1 response regarding stereotactic radiosurgery (SRS) and stereotactic body radiotherapy (SBRT) and ranked slight for 1 response regarding preparation for external beam radiotherapy. For the former, the relevant query was: “For SRS or SBRT, what will I feel during and after the procedure?” The LLM answered: “You will not feel any pain as it is non-invasive.” This was deemed to be harmful because it did not describe the invasive nature of SRS headframe placement if required. The expert answer noted possible pain associated with the placement of the headframe. The LLM-generated response rated as slight for potential harm pertained to the question: “Is there any special preparation needed for external beam therapy procedure.” The LLM did not note the need for tattoos at simulation.

Of 115 total questions retrieved from professional society websites, the LLM performed the same or better on 108 responses (94%) in relative correctness, 89 responses (77%) in completeness, and 105 responses (91%) in conciseness compared with expert responses (Figure 1B). For general radiation oncology answers, the LLM was rated as same, somewhat better, or much better in 100% for factual correctness, 90% for relative completeness, and 83% for relative conciseness (Figure 1C). The LLM treatment modality–specific answers were ranked the same or better for 91% of responses for relative factual correctness, 80% of responses for relative completeness, and 91% of responses for relative conciseness (Figure 1D). The LLM site-specific answers were ranked the same or better for 92% of responses for relative factual correctness, 66% of responses for relative completeness, and 98% of responses for relative conciseness (Figure 1E).

**Figure 2.**
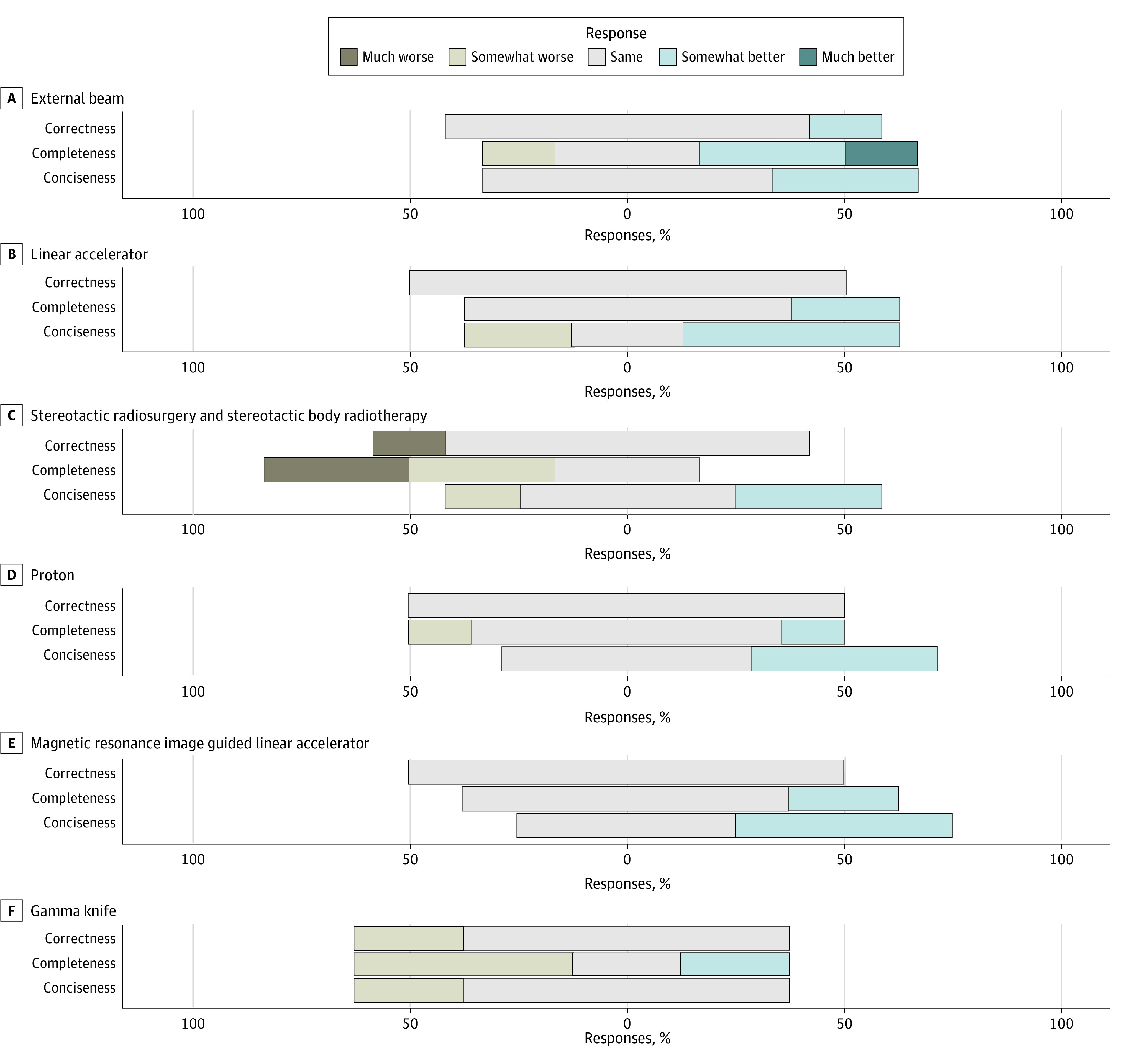
Relative Factual Correctness, Relative Completeness, and Relative Conciseness of Large Language Model (LLM)–Generated Responses in Each Subcategory Within Treatment Modality–Specific Answers Likert scale plot for relative factual correctness, relative completeness, and relative conciseness of LLM-generated responses compared with online resource expert answers in each subcategory within treatment modality–specific answers.

The treatment modality–specific answers encompassed 8 subcategories including external beam radiotherapy, linear accelerator, magnetic resonance imaging-guided linear accelerator (MR-LINAC), Gamma Knife, stereotactic radiosurgery (SRS), and stereotactic body radiotherapy (SBRT), intensity-modulated radiotherapy (IMRT), proton beam radiation therapy (PBT), and image-guided radiotherapy (IGRT). Within each category, the LLM was ranked as demonstrating the same, somewhat better, or much better conciseness for a range of 71% to 100% of questions; same, somewhat better, or much better completeness for 33% to 100% of questions; and same, somewhat better, or much better factual correctness for 75% to 100% of questions (Figure 2). Notably, the LLM responses related to “Gamma Knife” and “SRS and SBRT” had at least 50% of the LLM answers ranked as somewhat worse or much worse completeness than expert answers (Figure 2). Gamma Knife is the brand name for an SRS system, so the LLM responses appear to be less thorough and may omit important details when describing SRS and SBRT technologies (Figure 2). A complete ranking of all treatment modality–specific answers is presented in eFigure 1 in Supplement 1.

**Figure 3.**
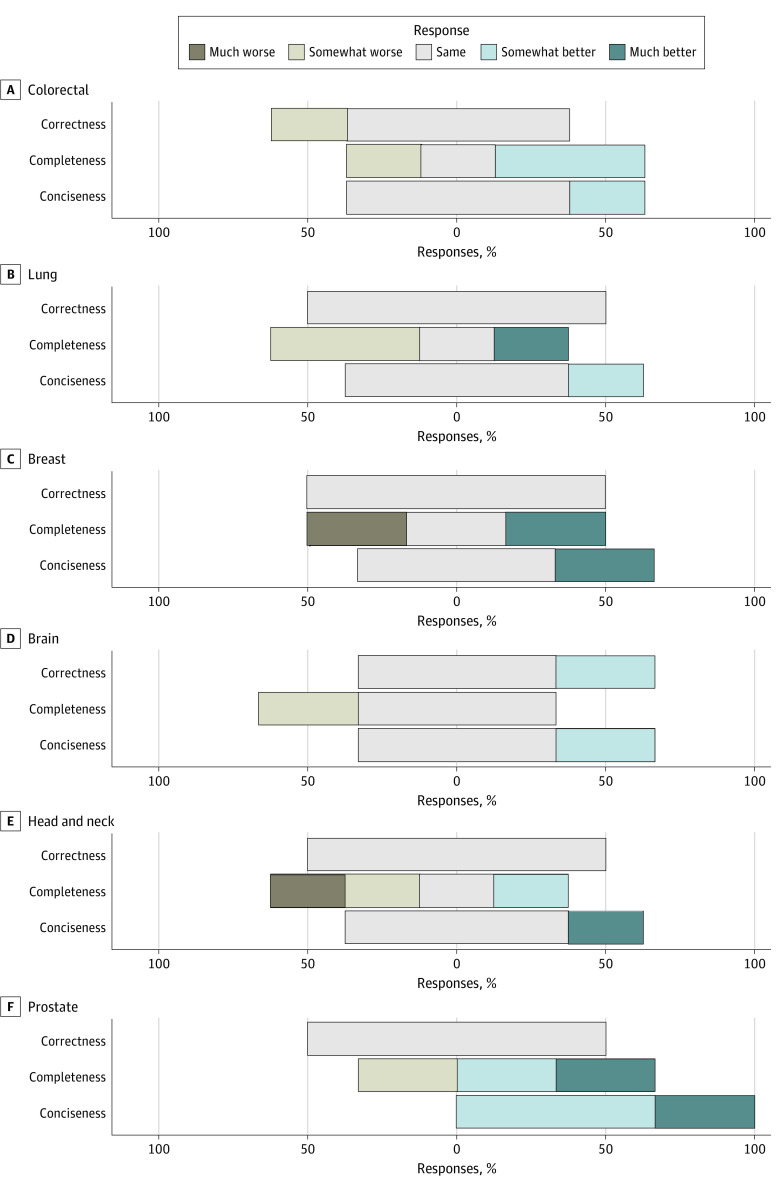
Relative Factual Correctness, Completeness, and Conciseness of Large Language Model (LLM)–Generated Responses Within Each Treatment Subsite–Specific Category Likert scale for relative factual correctness, completeness, and conciseness within each treatment subsite–specific category, covering colorectal, lung, breast, brain, head and neck, and prostate. Results for the remaining subsites are in eFigure 1 in Supplement 1.

Subsite-specific answers encompassed 11 subcategories, including colorectal, lung, breast, brain, head and neck, prostate, esophageal, pancreas, anal, gynecologic, and thyroid cancers. Within the subsites, the percentage of answers ranked as same, somewhat better, or much better ranged from 75% to 100% for relative factual correctness, 50% to 100% for relative completeness, and 75% to 100% for relative conciseness (Figure 3; eFigure 2 in Supplement 1). Cancer sites with the lowest ranked relative completeness included esophageal, lung, head and neck, and thyroid. For these subcategories, at least 50% of the LLM answers were ranked somewhat worse or worse than expert answers (Figure 3; eFigure 2 in Supplement 1).

**Figure 4.**
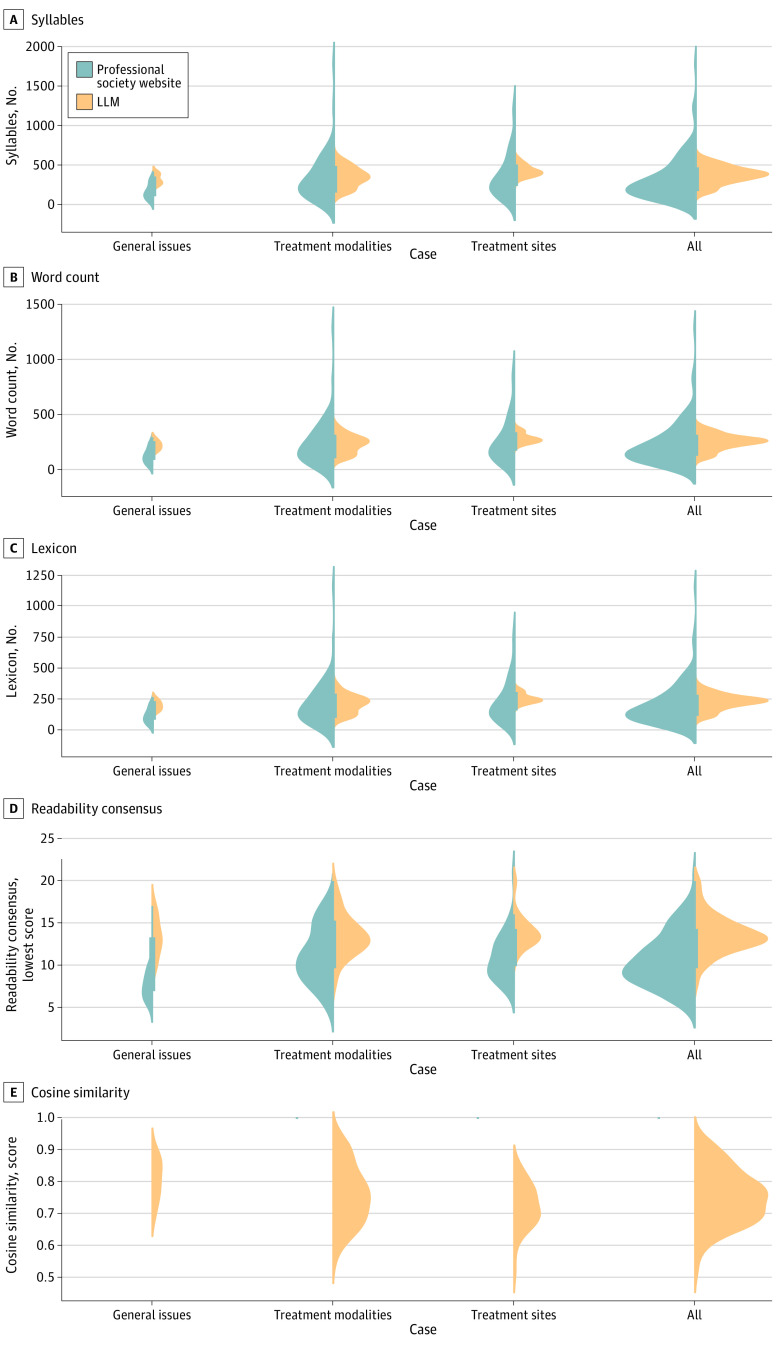
Computationally Generated Metrics for Large Language Model (LLM)–Generated Responses Computationally generated metrics for LLM-generated responses in categories of general radiation oncology issues, treatment modality–specific, and treatment site-specific.

Figure 4 presents distribution plots of domain-agnostic metrics, including syllable count, word count, lexicon scores, readability consensus, and cosine similarity. These metrics were computed for both the answers from professional society websites and the LLM responses, providing a comparative analysis. For all 115 questions, the mean (SD) number of syllables was 327.35 (277.15) for expert vs 376.21 (107.89) for LLM answers (*P* = .07), the mean (SD) word count was 226.33 (191.92) for expert vs 246.26 (69.36) for LLM answers (*P* = .27), and the mean (SD) lexicon score was 200.15 (171.28) for expert vs 219.10 (61.59) for LLM answers (*P* = .24) (Figure 4A, 4B, 4C). The mean (SD) readability consensus score was 10.63 (3.17) for expert answers, a 10th grade reading level, vs 13.64 (2.22) for LLM answers, a college reading level (*P* < .001) (Figure 4D). The mean (SD) cosine similarity between expert and LLM responses for all questions was 0.75 (0.09) (Figure 4E), where 1 is the highest possible similarity score, and 0 yields the lowest similarity between the expert responses and the LLM responses.

For general radiation oncology issues, the mean (SD) number of syllables was 164.20 (92.41) for expert vs 320.73 (63.23) for LLM answers (*P* < .001), the mean (SD) word count was 122.47 (66.86) for expert vs 215.53 (41.46) for LLM answers (*P* < .001), and the mean (SD) lexicon score was 106.20 (57.83) for expert vs 190.80 (38.37) for LLM answers (*P* < .001) (Figure 4A, 4B, 4C). The mean (SD) readability consensus scores in the grade level for expert and LLM answers were 7.47 (1.55) and 13.27 (2.28) (*P* < .001), indicating a 7th-grade reading level for expert answers and college reading level for LLM answers (Figure 4D). The mean (SD) cosine similarity of expert and LLM answers was 0.81 (0.06) (Figure 4E).

For treatment modality–specific answers, the mean (SD) number of syllables was 360.9 (308.1) for expert vs 361.9 (118.4) for LLM answers (*P* = .87), the mean (SD) word count was 247.5 (212.5) for expert vs 235.73 (76.35) for LLM answers (*P* = .77), and the mean (SD) lexicon score was 219.8 (191.1) for expert vs 211.13 (68.97) for LLM answers (*P* = .83) (Figure 4A, 4B, 4C). The mean (SD) readability consensus score in the grade level for expert vs the LLM was 11.27 (3.43) vs 13.49 (2.44) (*P* < .001), indicating an 11th-grade reading level for expert answers vs college reading level for the LLM (Figure 4D). The mean (SD) cosine similarity between expert and the LLM answers was 0.77 (0.09) (Figure 4E).

For site-specific answers, the mean (SD) number of syllables was 364.37 (275.83) for expert vs 428.8 (72.22) for LLM answers (*P* = .11), the mean (SD) word count was 251.29 (193.34) for expert vs 280.42 (45.6) for LLM answers (*P* = .27), and the mean (SD) lexicon score was 222.0 (170.8) for expert vs 248.47 (38.85) for LLM answers (*P* = .27) (Figure 4A, 4B, 4C). The mean (SD) readability consensus score in the grade level for expert and LLM answers was 11.0 (2.8) vs 13.93 (1.84) (*P* < .001), indicating an 11th-grade reading level for expert answers vs college reading level for LLM answers (Figure 4D). The mean (SD) cosine similarity between expert and LLM answers was 0.72 (0.07) (Figure 4E). For a more detailed exploration of these metrics, particularly focusing on the specific subcategories within treatment modality and treatment site answers, we have included eFigure 3 and eFigure 4 in Supplement 1. These supplementary figures offer a deeper insight into the nuanced performance of the LLM in these specialized areas, complementing the broader analysis presented in Figure 4.

## Discussion

To our knowledge, this study is one of the first to provide both domain-specific and domain-agnostic metrics to quantitatively evaluate ChatGPT-generated responses in radiation oncology. We have shown via both sets of metrics that the LLM yielded responses comparable with those provided by human experts via online resources and were similar, and in some cases better, than answers provided by the relevant professional bodies on the internet. Overall, the responses provided by the LLM were complete and accurate. Specifically, evaluators rated the LLM responses as demonstrating the same, somewhat better, or much better relative conciseness, completeness, and factual correctness compared with online expert resources for most answers.

Within the category of treatment modality, the LLM performed the worst with regard to potential harm and completeness for responses related to SRS (including Gamma Knife) and SBRT. SRS and SBRT are complex techniques utilized in radiation delivery, and the expert answers often included more detailed descriptions of the technology, how it is performed, indications, and the patient experience. The most notable omission by the LLM was the lack of mention of the SRS headframe. Headframe placement may be used during SRS and must be discussed with patients as the procedure is invasive and can be uncomfortable. The LLM responses related to SRS did not consistently mention the possibility of requiring a headframe, and if mentioned, did not describe potential discomfort or minor bleeding associated with headframe placement and removal.

The LLM-generated answers required a higher reading level than expert answers, with a large mean difference of 6 grade levels for the category of general radiation oncology answers, and a smaller mean difference of 2 grade levels for modality-specific and subsite-specific answers. The LLM generated more complex responses to the general radiation oncology questions, with higher mean syllable and word counts, and a higher mean lexicon score. These scores between expert and the LLM responses were similar for the modality-specific and site-specific answers. While these scores indicate more complex wording of the LLM responses to general radiation oncology questions, the syllable count, word count, and lexicon scores for the LLM responses were more consistent across answers in all 3 categories, with less variation across generated responses compared with expert answers.

Recommendations have been proposed to relieve the burden of physician inbox messages, including delegation of messages to other members of the team and charging payment for virtual communications; however, LLM chatbots are traditionally overlooked as a realistic solution.^35^ Concerns about accuracy and potential harm to patients have limited the use of LLM chatbots in the clinic; and ChatGPT’s creator, OpenAI, has acknowledged that the application may provide “plausible sounding but incorrect or nonsensical answers.”^35^ However, this study found high qualitative ratings of factual correctness as well as conciseness and completeness for the LLM answers to common patient questions. The LLM answers also had a high degree of similarity to expert answers, with a mean quantitative similarity score of 0.75.

### Limitations

This study had limitations. There are several limitations to both internet-based patient education materials and the LLM-generated responses. First, the high educational level required to understand the answers can be prohibitive. Thirteen online resource expert answers met the AMA and NIH recommendations for patient education resources to be written between third grade and seventh grade reading levels, whereas 0 LLM responses met the recommended reading level. Of the 119 LLM-generated answers, all except 14 responses were above a high school reading level. Many patients may have difficulty understanding the chatbot-generated answers, especially patients with lower health literacy and reading skills. Patients with lower reading skills are more likely to have poorer adherence to medications and overall poorer health, and patients with lower health literacy have more difficulties understanding their disease, radiation treatment, and potential side effects.^22^

Despite these limitations, a unique capability of the LLM is the ability to generate tailored responses through specific prompts. Directed prompts such as “Explain this to me like I am in fifth grade” may help generate simplified responses. Techniques such as 1-shot or few-shot prompting can potentially enhance the model’s reasoning, a prospect for future exploration. Although obtaining an optimized response might require multiple prompts, the LLM offers a more convenient alternative to consulting various online resources. In developing our study’s query database, we found no single online resource that comprehensively addressed common patient questions.

Second, individual variations in question phrasing could affect the evaluated metrics such as conciseness, completeness, and factual correctness in our study. The LLM responses might differ based on each user’s background, language proficiency, and comfort with technology. Furthermore, as experimental models such as the LLM evolve with ongoing user interactions and data updates, responses to the same query may change over time. Thus, continuous monitoring and updating of chatbot iterations are essential. While the LLM shows great potential to augment clinician-patient interactions, further work on the effect on clinic efficiency and qualitative measures of patient satisfaction with the incorporation of the LLM into clinic workflows should be explored.

Although our study primarily uses ChatGPT, one of the earliest publicly released LLMs, other models such as Bard (Google), LLAMA (Meta), and Claude (Anthropic) also show promise in addressing radiation oncology queries. These LLMs differ in capabilities: Bard is known for creative content and current data, LLAMA for its customization in noncommercial applications, and Claude for prioritizing safe and ethical AI interactions.^36^ Exploring these diverse LLMs using our study’s framework could provide further insights into their effectiveness in radiation oncology and medical communication.

## Conclusion

This cross-sectional study found that the LLM provided mainly highly accurate and complete responses in a similar format to virtual communications between a patient and clinicians in a radiation oncology clinical environment. Accordingly, these results suggest that the LLM has the potential to be used as an alternative to current online resources.

## Notes

Supplement 1. **eFigure 1.** A Complete Ranking of All Treatment Modality-Specific Answers **eFigure 2.** Likert Scale Comparison of ChatGPT-Generated Responses and Online Resource Expert Answers **eFigure 3.** Computationally Generated Metrics for ChatGPT Generated Responses in Each Subcategory Within Treatment Modality-Specific Answers **eFigure 4.** Computationally Generated Metrics for ChatGPT Generated Responses in Each Subcategory Within Treatment Subsite-Specific Answers

Supplement 2.
Data Sharing Statement
