# Supplementary material for: Quality of Large Language Model Responses to Radiation Oncology Patient Care Questions
Source: JAMA Netw Open. 2024 Apr 2;7(4):e244630. doi: 10.1001/jamanetworkopen.2024.4630 (PMC10988356; doi:10.1001/jamanetworkopen.2024.4630)
Supplement: Supplement 1. — eFigure 1. A Complete Ranking of All Treatment Modality-Specific Answers eFigure 2. Likert Scale Comparison of ChatGPT-Generated Responses and Online Resource Expert Answers eFigure 3. Computationally Generated Metrics for ChatGPT Generated Responses in Each Subcategory Within Treatment Modality-Specific Answers eFigure 4. Computationally Generated Metrics for ChatGPT Generated Responses in Each Subcategory Within Treatment Subsite-Specific Answers [file jamanetwopen-e244630-s001.pdf]

Supplemental Online Content

Yalamanchili A, Sengupta B, Song J, et al. Quality of large language model responses to radiation oncology patient care questions. *JAMA Netw Open*. 2024;7(4):e244630. doi:10.1001/jamanetworkopen.2024.4630

eFigure 1. A Complete Ranking of All Treatment Modality-Specific Answers

eFigure 2. Likert Scale Comparison of ChatGPT-Generated Responses and Online Resource Expert Answers

eFigure 3. Computationally Generated Metrics for ChatGPT Generated Responses in Each Subcategory Within Treatment Modality-Specific Answers

eFigure 4. Computationally Generated Metrics for ChatGPT Generated Responses in Each Subcategory Within Treatment Subsite-Specific Answers

This supplemental material has been provided by the authors to give readers additional information about their work.

© 2024 Yalamanchili A et al. *JAMA Network Open.*

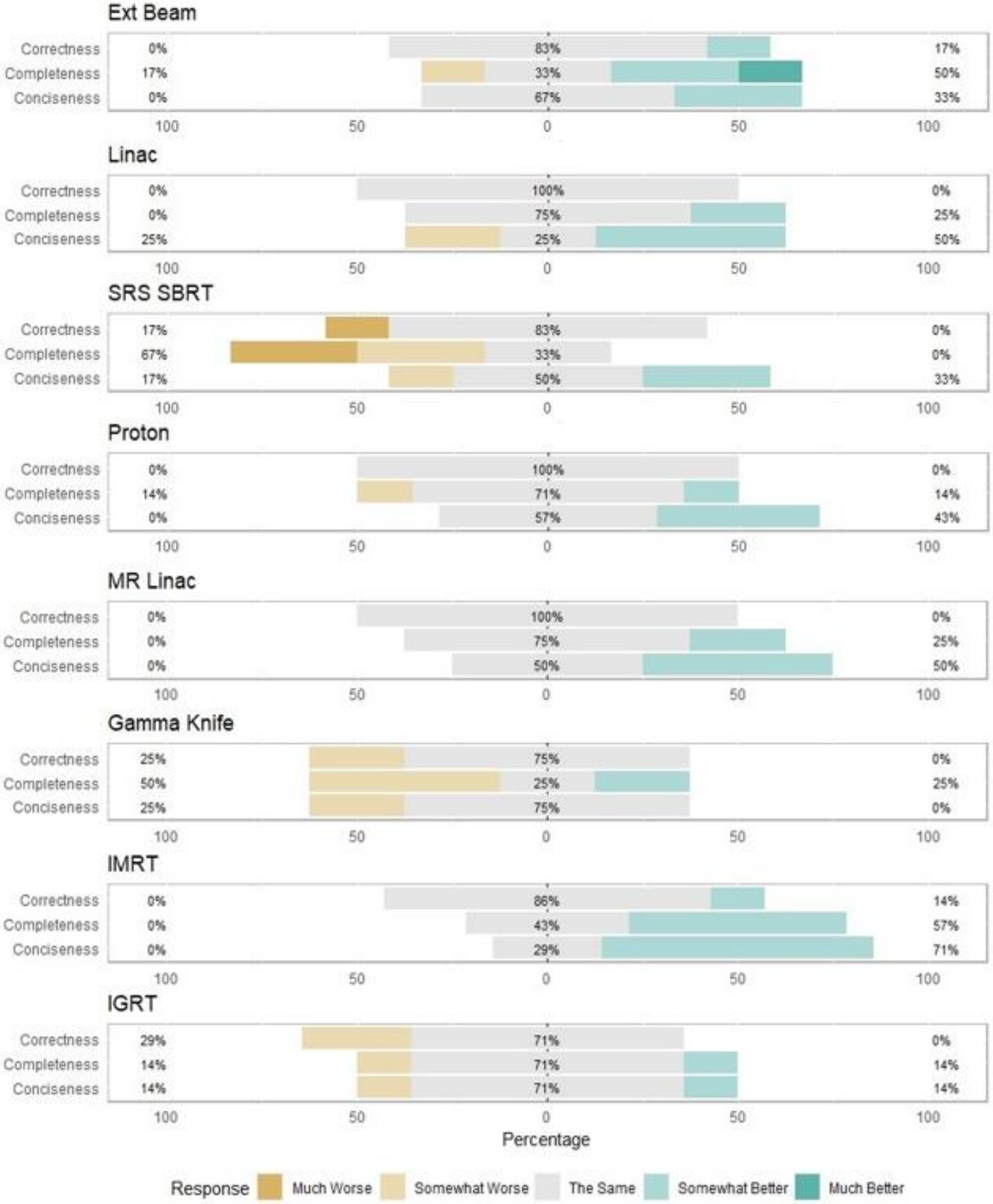

eFigure 1: A complete ranking of all treatment modality-specific answers (a continuation of Figure 2).

© 2024 Yalamanchili A et al. *JAMA Network Open.*

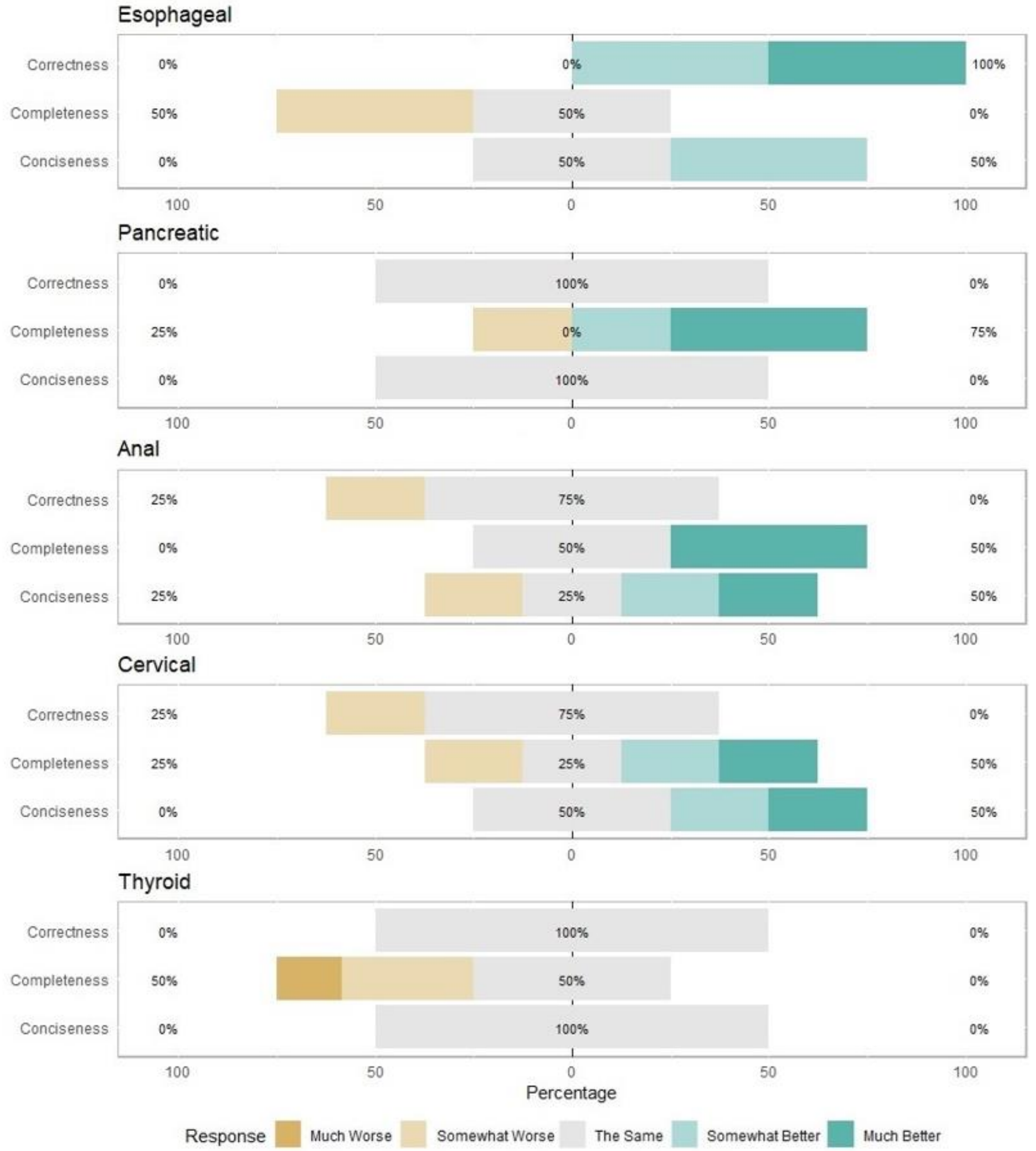

eFigure 2. Continuation of Figure 3 – Likert scale comparison of ChatGPT-generated responses and online resource expert answers. This plot illustrates the relative factual correctness, completeness, and conciseness within each treatment subsite-specific category, covering esophageal, pancreatic, anal, cervical, and thyroid cancers.

© 2024 Yalamanchili A et al. *JAMA Network Open.*

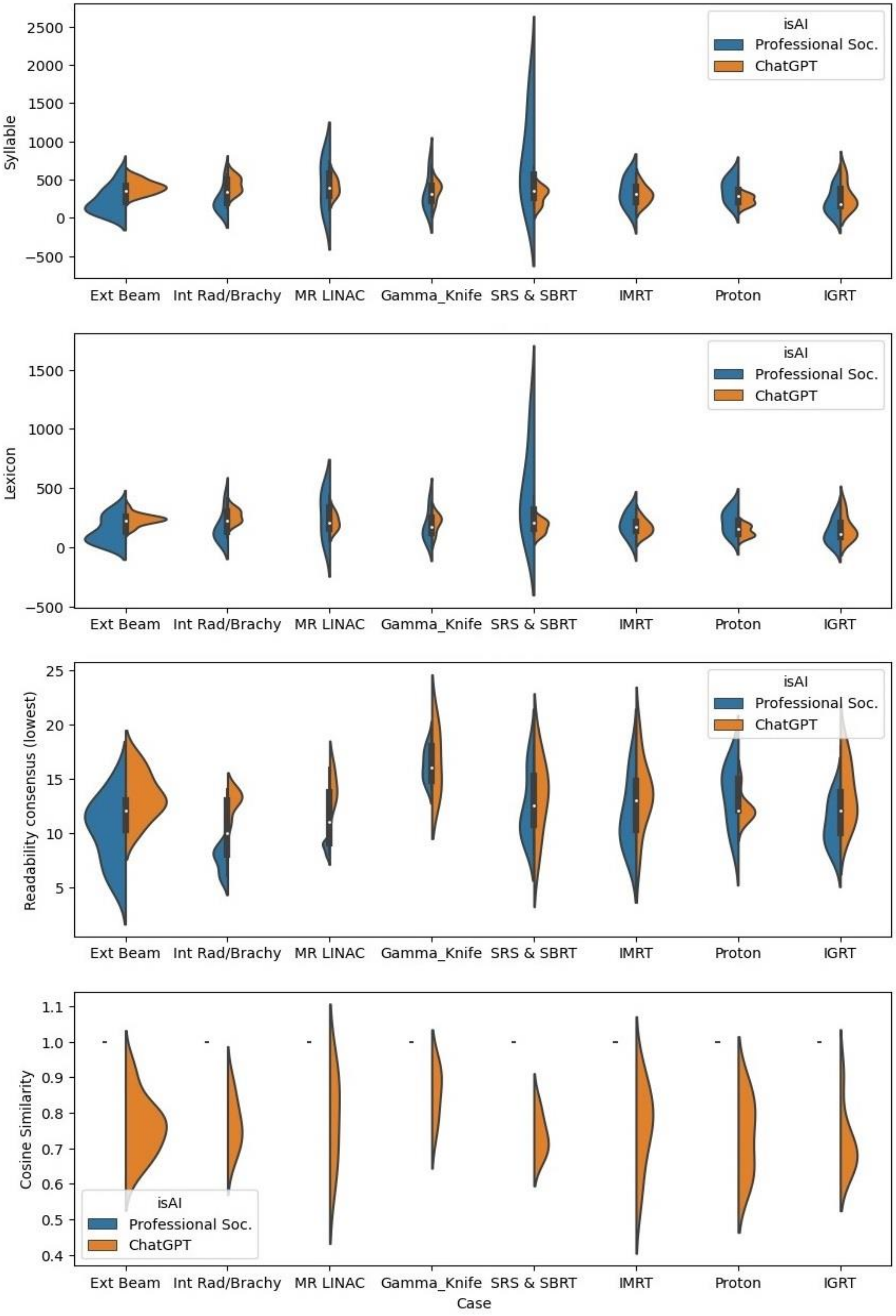

eFigure 3. Computationally generated metrics for ChatGPT generated responses in each subcategory within treatment modality-specific answers.

© 2024 Yalamanchili A et al. *JAMA Network Open.*

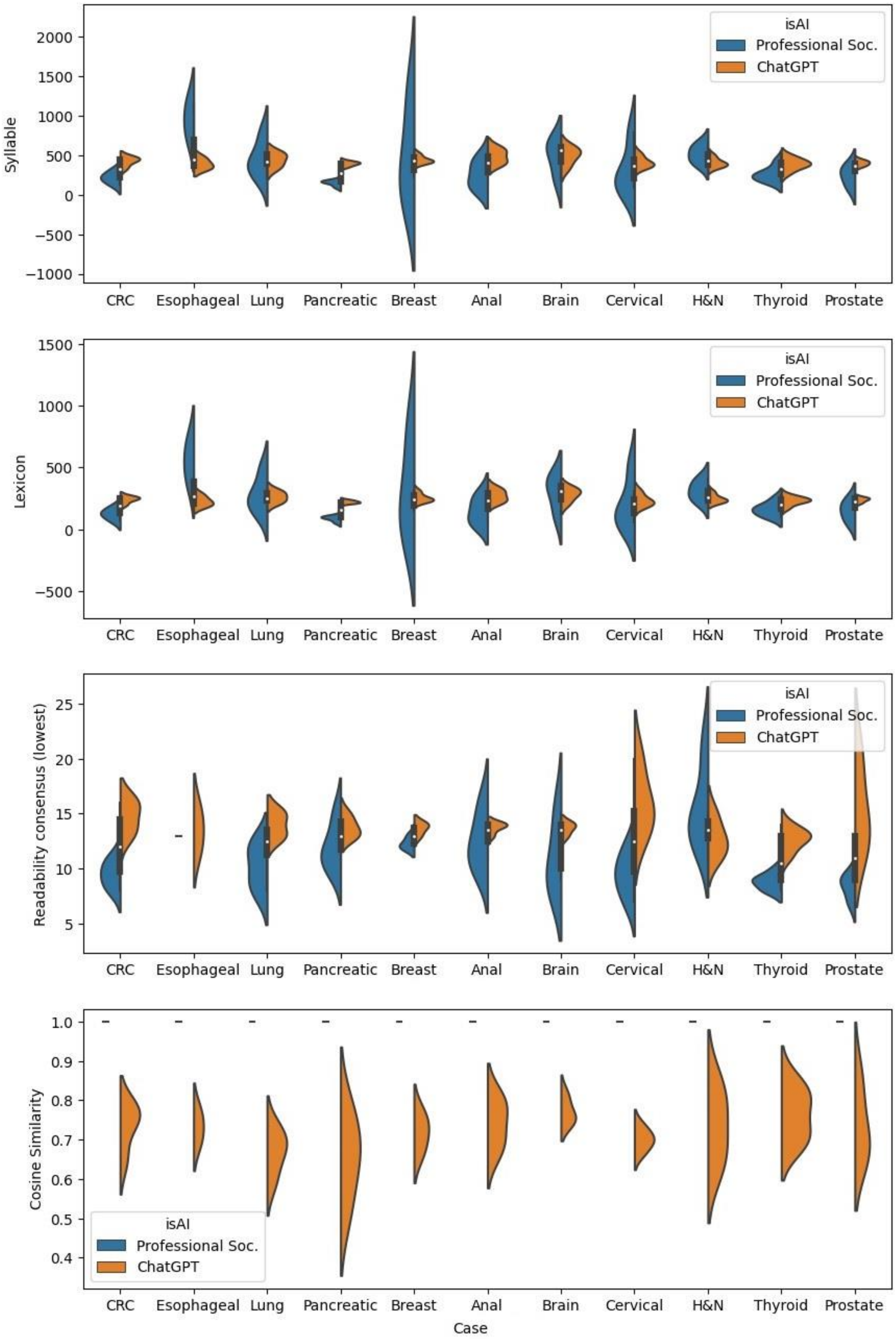

eFigure 4. Computationally generated metrics for ChatGPT generated responses in each subcategory within treatment subsite-specific answers.

© 2024 Yalamanchili A et al. *JAMA Network Open.*
