# Supplementary material for: Quality of Large Language Model Responses to Radiation Oncology Patient Care Questions
Source: JAMA Netw Open. 2024 Apr 2;7(4):e244630. doi: 10.1001/jamanetworkopen.2024.4630 (PMC10988356; doi:10.1001/jamanetworkopen.2024.4630)
Supplement: Supplement 2. — Data Sharing Statement [file jamanetwopen-e244630-s002.pdf]

Data Sharing Statement

Yalamanchili. Quality of Large Language Model Responses to Radiation Oncology Patient Care Questions. *JAMA Netw Open*. Published April 02, 2024.
doi:10.1001/jamanetworkopen.2024.4630

Data
Data available: No
